# Effects of Methionine Restriction from Different Sources on Sperm Quality in Aging Mice

**DOI:** 10.3390/nu15224782

**Published:** 2023-11-15

**Authors:** Yinghui Wu, Hao Li, Yueyue Miao, Jian Peng, Hongkui Wei

**Affiliations:** 1 Department of Animal Nutrition and Feed Science, College of Animal Science and Technology, Huazhong Agricultural University, Wuhan 430070, China; wuyinghui@mail.hzau.edu.cn (Y.W.); miaoyueyue@mail.hzau.edu.cn (Y.M.); pengjian@mail.hzau.edu.cn (J.P.); 2 The Cooperative Innovation Center for Sustainable Pig Production, Wuhan 430070, China; 3 Frontiers Science Center for Animal Breeding and Sustainable Production, Wuhan 430070, China

**Keywords:** aging mice, methionine hydroxy analog, methionine restriction, methylation, sperm quality, spermidine

## Abstract

Decreased sperm quality causing poor pregnancy outcomes in aging males is a common problem. The aim of this study was to investigate the ameliorative effect of methionine restriction on sperm quality in aging mice, using methionine or 2-hydroxy-4-(methylthio)butanoate (HMTBA) as the methionine source, with a view to providing nutritional strategies to mitigate the decline in sperm quality in aging livestock. Fifty-one 6-week-old male mice were randomly divided into four groups: the non-aging group (NA, 0.86% methionine), the control diet group (CD, 0.86% methionine), the methionine-restricted group (MR, 0.17% methionine) and the HMTBA-restricted group (HR, 0.17% methionine). The mice in the CD, MR and HR groups were injected with a daily dose of 0.25 mL/20 g body weight of 10% D-galactose to establish an aging model. The test period was 42 days. The results showed that aging mice in the CD group had impaired testicular morphology and significantly decreased sperm quality compared to those in the NA group. Aging mice in the MR and HR groups showed attenuated impaired testicular morphology and improved sperm quality, especially sperm acrosomal integrity and membrane integrity, compared to mice in the CD group. In addition, mice in the MR and HR groups had reduced testicular inflammation and oxidative stress, increased spermidine levels, and reduced sperm RNA N6-methyladenosine (m^6^A) and DNA 5-methylcytosine (5mC) levels. Spermidine levels were positively correlated, whereas sperm RNA m6A and DNA 5mC levels were negatively correlated with sperm quality parameters. Our study suggests that methionine restriction alleviates the decline in sperm quality in aging mice, which may be related to changes in methionine metabolism and inhibition of sperm DNA and RNA methylation.

## 1. Introduction

The phenomenon of delayed parenthood in the population due to socio-economic factors and the increasing age of livestock and poultry in intensive farms due to prolonged stock value is increasingly common, especially for males [1]. Therefore, it is particularly important to maintain superior sperm quality and fertility in older male animals. However, recent studies have indicated that, as males age, there is a decrease in sperm motility [2,3], an increase in abnormal sperm rate [2,3] and sperm deoxyribonucleic acid (DNA) damage [4] and poorer pregnancy outcomes [5]. These manifestations may be associated with increased levels of inflammatory factors [6] and oxidative stress [7], as well as age-related changes in tissue-specific methylation status [8,9,10]. Aging has been shown to hypermethylate ribosomal DNA in rat sperm [11], and the production of defective sperm has been closely linked to global DNA hypermethylation [12]. Therefore, it is of great significance to study nutrition strategies to delay the decline of sperm quality caused by the aging of male animals to improve the adverse consequences of delayed parenthood and increase the life and value of livestock and poultry breeding.

Methionine, an amino acid necessary for the normal growth and development of many organisms, is not only a key methyl source for methylation reactions but also contributes to the production of important metabolites such as polyamines, nucleotides and glutathione. However, the benefits of methionine restriction, while maintaining total energy intake, on the physiological functions of the organism have been widely discussed. For example, the normal basal diet of mice contains 0.86% methionine [13]. Reducing the methionine content by 80% (0.17% methionine) has been shown to extend the lifespan in Drosophila and rodents [14], which may be related to the modulation of aging by methionine restriction and its ability to inhibit DNA methylation [15,16]. In addition, although methionine restriction may reduce glutathione production in some tissues, it does not affect glutathione synthesis capacity [17] but instead increases levels of antioxidant enzymes, thereby reducing oxidative stress and inflammation and improving physiological function in senescent animals [18,19]. This effect may be closely related to the promotion of hydrogen sulfide production when methionine is restricted [19,20]. It follows that methionine restriction may be a feasible nutritional strategy to prevent and improve aging-related diseases and functional impairments in the future. However, the effect of methionine restriction on sperm quality decline in aging animals remains unclear.

L-methionine and methionine hydroxy analogs are commonly used as methionine additives in livestock and poultry production. However, it is worth noting that different sources of methionine have different metabolic capacities and effects on DNA and ribonucleic acid (RNA) methylation modification. Previous studies have shown that methionine hydroxy analogs, such as 2-hydroxy-4-(methylthio) butanoic acid (HMTBA), can escape more first-pass metabolism in the gut, increase the content of metabolites in other tissues [21] and significantly reduce the level of N6-methyladenosine (m^6^A) in RNA in intestinal epithelial cells compared to L-methionine [22]. RNA m^6^A is the most common and conserved mRNA modification in eukaryotes and regulates gene expression by affecting RNA stability, translocation, translation and degradation. Many studies have confirmed that m^6^A methylation regulates several important physiological processes, such as autophagy, inflammation, oxidative stress and DNA damage, during aging [23]. Therefore, it is worth exploring whether methionine restriction from different sources has the same alleviating effect on sperm quality decline in aging male animals.

Based on the problem of sperm quality reduction caused by aging, this study established an aging mouse model to explore the improvement effect of L-methionine and HTMBA methionine restriction on the sperm quality of aging mice and the regulatory mechanisms of sperm DNA and RNA methylation.

## 2. Materials and Methods

### 2.1. Animal Procedures, Diets and Treatments

Fifty-one healthy C57BL/6 male mice, 6 weeks old and weighing 18–20 g, were acquired from the Animal Experiment Centre of Huazhong Agricultural University (Wuhan, China). The mice were randomly divided into four groups according to the principle of non-preference: the non-aging group (NA, *n* = 12, 21.45 ± 0.57 g), the control group (CD, *n* = 13, 21.26 ± 0.39 g), the methionine-restricted group (MR, *n* = 13, 21.09 ± 0.44 g) and the HMTBA-restricted group (HR, *n* = 13, 21.14 ± 0.48 g). There was no difference in the initial health status, age and body weight of the mice in the four groups. Mice in the CD, MR and HR groups were injected with 0.25 mL/20 g body weight of 10% D-galactose behind the neck every day from 9:00 to 11:00 to establish a D-galactose-induced aging mouse model [24], and mice in the NA group were injected with an equal amount of phosphate-buffered solution every day until the end of the test. The methionine restriction test was performed using 80% methionine restriction, and mice in the NA, CD, MR and HR groups were fed diets containing 0.86% methionine, 0.86% methionine, 0.17% methionine and 0.17% HMTBA, respectively. The experimental diets were purchased from Trophy Company (Nantong, China), and the specific formulations are listed in Table 1. The experiment lasted 42 d, during which time the mice were housed in identical cages of the same size, with 4–5 mice in each cage. Cages were numbered, different mice in the same cage were distinguished by numbers, and all cages were placed on two adjacent levels in the middle section of the same shelf. The grouping of the mice was not known to the feeder. Mice were housed under specific pathogen-free, 12 h light/dark, 21 ± 3 °C and 55 ± 10% humidity conditions. Food and drinking water were provided ad libitum. At the end of the test, the mice were marked with the cage number and the number of each mouse in the cage (e.g., mouse 1-1), and the results of each indicator were assessed using the same methods of slaughter, sampling and indicator testing. Situations such as outliers in a mouse’s results that may occur during the indicator testing process were dealt with by re-sampling and re-testing.

All procedures were approved by the Ethics Committee of Huazhong Agricultural University (IACUC number: HZAUMO-2022-0201), which complied with animal biomedical research principles formulated by the China Animal Care Committee and the Council of the International Medical Organization.

### 2.2. Blood and Tissue Preparation

After the end of the trial period, all mice were sacrificed by decapitation after blood collection from the eyeballs. Serum was obtained after centrifugation of the blood at 4000 rpm (Centrifuge 5810R, Eppendorf, Hamburg, Germany) for 10 min at 4 °C and stored at −80 °C. The testes and epididymides were collected, and the right testes of the mice were taken and fixed in 4% paraformaldehyde solution for morphological analysis. The rest of the testes were first frozen in a −20 °C freezer and then transferred to a −80 °C ultra-low temperature freezer for longer storage. The bilateral epididymides were immediately put in 2 mL of prewarmed saline solution (37 °C) and then cut into pieces to prepare a sperm suspension for subsequent determination.

### 2.3. Measurement of Body Weight and Feed Intake

Feed intake of mice in each group was measured daily; the body weight of mice in each group was measured every week. Feed intake was calculated as follows: feed intake = initial feed weight − remaining feed weight the next day. The average daily feed intake (g/d) and average daily weight gain (g/d) were calculated for the 42 days.

### 2.4. Testicular Histopathology Analysis

The paraformaldehyde-fixed mouse testes were trimmed, rinsed, dehydrated, pierced, immersed in wax, embedded and sectioned. Hematoxylin-eosin staining was used to stain and seal the testes, and changes in testicular histology were observed and compared under light microscopy.

### 2.5. Determination of Serum Testosterone Content

The serum testosterone content of mice was determined using the Mouse Testosterone Elisa Assay Kit Instruction (Nanjing Jiancheng Bioengineering Institute, Nanjing, China). Briefly, add serum samples to enzyme well that has been pre-coated with antibodies, then add recognition antigen labeled by horse radish peroxidase (HRP) and incubate at 37 °C for 30 min, followed by five washes. Add affinity hormone HRP, incubate for 30 min and wash. Subsequently, add the chromogenic solution and incubate for 10 min away from light, and then add the stop solution. The OD value of the serum samples was detected at 450 nm, and the testosterone concentration in the samples was calculated according to the logistic curve obtained by fitting the testosterone standard solution.

### 2.6. Determination of Sperm Quality

Sperm concentration: One side of the epididymis was taken and placed in 1 mL of prewarmed normal saline (37 °C). It was cut into pieces and prepared as a sperm suspension. The suspension was then placed in a 37 °C water bath and incubated for 20 min. After mixing well, 10 μL of the suspension was taken and placed into a prewarmed hemocytometer. According to the red blood cell counting method, we counted the total number of sperm in 5 squares and multiplied the total number of sperm by 100,000, which is the total number of sperm in the sample, expressed as 10^6^ sperm/mL. 

Sperm motility: A drop of 10 μL of sperm suspension was dropped into a prewarmed hemocytometer at 37 °C. Active sperm, i.e., the percentage of sperm moving in a straight line in the field of view of the microscope, were counted from 200 sperm under high magnification. Sperm motility = number of sperm in a straight line/total sperm × 100%. Each sample was tested 2 times and the average was recorded.

Abnormal sperm rate: A total of 10 μL of sperm suspension was aspirated onto a slide, blotted and air-dried. After drying, the slides were treated with methanol for 15 min, stained with 2% eosin for 1 h, rinsed with ultrapure water, dried and examined morphologically [25]. At least 200 sperm per mouse were observed under a high-power microscope, and the presence of head deformity, mid-end fracture, tail deformity or protoplasmic droplets was recorded as abnormal sperm. The abnormal sperm rate was calculated using the formula: Abnormal sperm rate = number of abnormal sperm/total sperm × 100%. Each sample was tested 2 times and the average was recorded.

Sperm acrosome integrity and sperm membrane integrity: A 100 μL aliquot of sperm suspension sample was placed in a glass slide; Giemsa staining was performed after the samples were fixed with 4% formaldehyde. Acrosome staining of 200 sperm from 5 different regions was observed by using light microscopy under 400× magnification to calculate acrosome integrity. Subsequently, another sperm suspension of 100 μL was taken, and the sperm membrane integrity was detected by hypotonic swelling method. Each sample was tested 2 times and the average was recorded.

### 2.7. Determination of Methionine Metabolites in Testis

The testicular methionine metabolite concentrations were determined with reference to our previous study [26]. Briefly, testis was first prepared as a 10% tissue homogenate solution using KH_2_PO_4_ solution, followed by centrifugation at 13,000× *g*/min for 5 min, and the supernatant was collected for analysis of methionine and its metabolites. The protein content of the samples was also determined using the bicinchoninic acid assay (BCA) kit to calculate the metabolite content of the samples. Subsequently, 20 μL of the supernatant was added to 80 μL of tris (2-carboxyethyl) phosphine solution and incubated for 10 min at room temperature, 400 μL of methanol solution containing 1% formic acid was added, vortexed for 10 s and then incubated at −20 °C for 2 h. The samples were centrifuged at 13,000 rpm for 10 min at 4 °C, and the supernatants were extracted and filtered through 0.22 μm filter. Liquid chromatography–tandem mass spectrometry (LC-MS/MS) analysis was performed on an LCMS-8050 triple quadrupole mass spectrometer (Shimadzu, Kyoto, Japan) equipped with an LC-30AD system (Shimadzu, Kyoto, Japan) and a SIL-30AD autosampler (Shimadzu, Kyoto, Japan). The chromatographic separation was performed on a UPLC XSelect High Strength Silica T3 1.8 μm, 100 × 2.1 mm I.D. column (Waters, Milford, MA, USA) with gradient elution. The sample temperature in the autosampler was maintained at 4 °C, and the sample volume was 1 μL per injection.

The spermidine content of testis was measured using the Mouse Spermidine Detection Kit (Nanjing Jiancheng Bioengineering Institute, Nanjing, China). The testes were first prepared as a 10% tissue homogenate using saline solution, followed by centrifugation at 3000× *g*/min for 10 min, and the supernatant was collected for analysis of spermidine content. The protein content of the samples was also determined using the BCA kit to calculate the spermidine content of the samples. Samples were added to enzyme wells pre-coated with antibodies, then HRP-labeled recognition antigen was added and incubated at 37 °C for 60 min, followed by five washes. The chromogenic solution was then added and incubated for 15 min in the dark, and the stop solution was added. The optical density (OD) value of the samples was detected at 450 nm, and the spermidine content in the samples was calculated according to the linear standard curve obtained by fitting the spermidine standard solution.

### 2.8. Testicular RNA Extraction, Reverse Transcription and Real-Time Fluorescence Quantitative PCR

Testes were ground in liquid nitrogen, and total RNA was then extracted by TRIzol. The purity and concentration of the extracted RNA were determined using Nanodrop ultrafine spectrophotometer. After RNA extraction, samples should have had the required RNA purity with a 260/280 ratio between 1.8 and 2.0. Samples with a 260/280 ratio less than 1.8 were subjected to RNA re-extraction and subsequent analysis after the purity requirement was met. Total RNA was stored at −80 °C until used for cDNA synthesis. The reverse transcription reaction was performed using the reverse transcription kit according to the manufacturer’s instructions. Reverse transcription quantitative polymerase chain reaction (RT-qPCR) was performed using the cycling in the fluorescence excitation (CFX) real-time PCR detection system, and the PCR reaction conditions were as follows: pre-denaturation at 95 °C for 5 min; 95 °C for 10 s; annealing for 10 s; and extension for 30 s, for a total of 40 cycles. The fluorescence signal was collected once at the end of each cycle, and the lysis curve was analyzed at the end of the reaction. The primer efficiency of each gene was within an acceptable range (90–110%), and the primer sequences are shown in Table 2. Finally, GAPDH was used as an internal reference gene for each target gene, and the relative level of the target genes was calculated by the 2^−ΔΔCt^ method [27].

### 2.9. Determination of DNA and RNA Methylation Levels in Sperm

RNA methylation levels in sperm: The sperm RNA m^6^A methylation level was detected by LC-MS/MS method [26]. Initially, sperm samples collected from the epididymis were rinsed 3 times with PBS, sperm mRNA was purified using an mRNA extraction kit and the sperm cap structure was removed using RppH enzyme (NEB.M0356S). The samples were then cleaved into ribonucleotides using Nuclease S1. The samples were then treated with 1 mol/L NH_4_HCO_3_ and alkaline phosphatase at 37 °C for 2 h. Finally, the digestion products were added to 200 μL of ultrapure water, filtered through 0.22 μm and loaded into clean liquid chromatography vials, and 1–5 μL of the above liquid was taken for LC-MS/MS. For sample detection, the standard curves of A and m^6^A were constructed by using the standards of A and m^6^A, and the measured values of the samples were fitted to the standard curves to obtain the contents of m^6^A and A. The ratio of m^6^A to A was used to calculate the amount of m^6^A in the sample.

DNA methylation levels in sperm: Sperm samples collected from mouse epididymis were washed with PBS for 3 times, and then total sperm DNA was extracted with DNA extraction kit. DNA 5-Methylcytosine (5mC) methylation levels in sperm were measured using a DNA methylation test kit (EPT-P-1034-48, EpiGentek, Shanghai, China). In this assay, a prepared genomic DNA sample was added to strip wells that are specifically treated to have a high DNA affinity. The methylated fraction of DNA was detected using capture and detection antibodies and then quantified colorimetrically by reading the absorbance in a microplate spectrophotometer. The amount of methylated DNA is proportional to the OD intensity measured.

### 2.10. Statistical Analysis

For the statistical analysis of the parameters, the Shapiro–Wilk test was used to check for normality and to verify that the average daily feed intake, serum testosterone levels, sperm concentration, sperm motility, abnormal sperm rate, effective sperm count, sperm acrosome integrity and membrane integrity presented non-normal distribution. The remaining indicator data were normally distributed or transformed to a normal distribution by square root or natural ln transformation. As the results of the measured parameters presented normal and non-normal distributions, the results within the same groups were presented as median (quartile 1–quartile 3) (non-normal distribution) or mean ± SD (normal distribution, including square root and ln normal distribution). The significance of the difference between groups was calculated using one-way ANOVA (normal distribution) or Wilcoxon signed-rank test (non-normal distribution). Pearson’s correlation coefficients (normal distribution) or Spearman’s rank (non-normal distribution) were used to determine the correlation between sperm quality parameters and methionine metabolite levels and sperm RNA m^6^A and DNA 5mC levels. Data were statistically analyzed using SAS 8.2 software (SAS Inst., Inc., Cary, NC, USA) and plotted using GraphPad Prism 8.0 (GraphPad Software, La Jolla, CA, USA). For all analyses, *p* < 0.05 indicated a significant difference, and *p* < 0.01 indicated an extremely significant difference.

## 3. Results

### 3.1. Growth Performance of Aging Mice with Methionine Restriction

**Figure 1:**
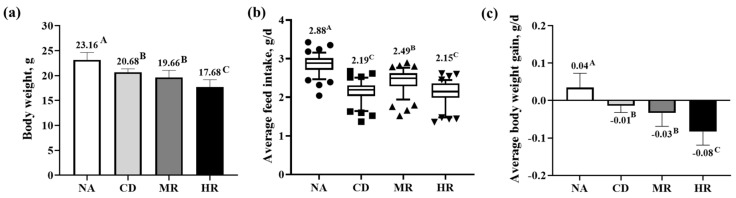
Growth performance. (**a**) Body weight of the mice at the end of the trial period; (**b**) Average daily feed intake of mice in 0–42 d; (**c**) Average daily gain of mice in 0–42 d. Data for body weight and average daily gain conformed to a normal distribution and are expressed as mean ± standard deviation (SD); data for average daily feed intake did not conform to a normal distribution and are expressed as median and 10–90% percentile (NA group, *n* = 12; CD, MR and HR groups, *n* = 13). In the same figure, values with different capital letter superscripts indicate extremely significant differences (*p* < 0.01).

The body weight (*p* < 0.01), average daily feed intake (*p* < 0.01) and daily gain (*p* < 0.05) of the aging mice in the CD group were significantly lower than those of the normal mice in the NA group. Compared with the CD group, the average feed intake of aged mice in the MR group was increased, but the average daily gain was decreased; the body weight and average daily gain of mice in the HR group were significantly decreased. HR treatment inhibited the body weight, average daily feed intake and daily gain of aging mice to a greater extent than MR treatment (Figure 1).

### 3.2. Testicular Histopathology Analysis and Sex Hormone Levels of Aging Mice with Methionine Restriction

**Figure 2:**
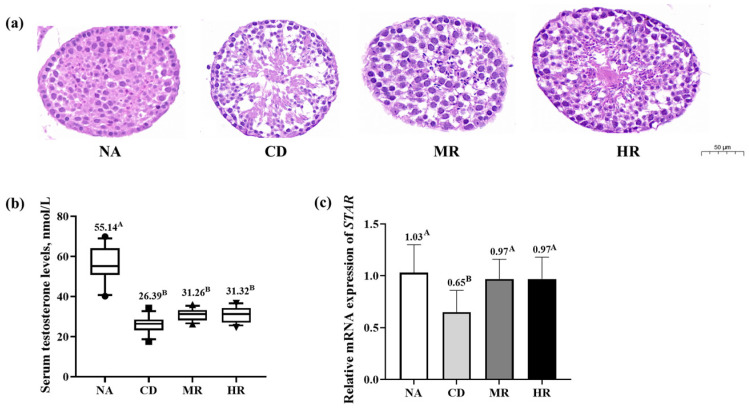
Testicular histopathology analysis and sex hormone levels. (**a**) Testicular microstructure. The scale bar is 50 μm, which is a 20× magnification mirror, and the red area is a 100× magnification mirror; (**b**) Serum testosterone content. Data are presented as median and 10–90% percentile (NA group, *n* = 12; CD, MR and HR groups, *n* = 13); (**c**) Relative mRNA expression of steroidogenic acute regulatory protein (*STAR*) in testes. Data are presented as the mean ± SD (NA, CD, MR and HR groups, *n* = 8). In the same figure, values with different capital letter superscripts indicate extremely significant differences (*p* < 0.01).

In the CD group, the structure of the seminiferous tubules in the testes was loose, the spermatogenic cells were shed, and the serum testosterone level (*p* < 0.01) and the relative mRNA expression of the testosterone synthase-related protein *STAR* (*p* < 0.01) were significantly lower than those of normal mice in the NA group. The shedding of spermatogenic cells in the testes of aging mice in the MR and HR groups was slightly better than that in the CD group, and the *STAR* expression was significantly higher than that in the CD group (*p* < 0.01). However, there was no significant difference in testosterone levels and *STAR* expression between the MR and HR groups (*p* > 0.05, Figure 2). This suggests that the impaired testicular histology and sex hormone synthesis in aging mice could be alleviated by methionine restriction.

### 3.3. Sperm Quality of Aging Mice with Methionine Restriction

**Figure 3:**
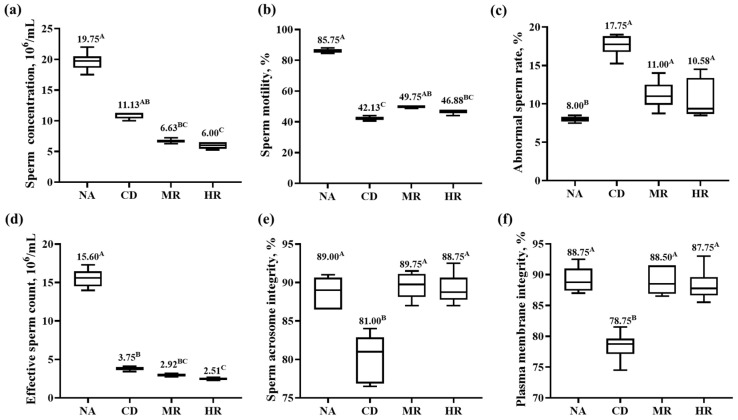
Sperm concentration and sperm quality. (**a**) Sperm concentration in mouse epididymis; (**b**) Sperm motility; (**c**) Abnormal sperm rate; (**d**) Effective sperm count; (**e**) Sperm acrosome integrity; (**f**) Sperm membrane integrity. Data are presented as median and 10–90% percentile (NA, CD, MR and HR groups, *n* = 6). In the same figure, values with different capital letter superscripts indicate extremely significant differences (*p* < 0.01).

Compared with the NA group, the sperm motility, effective sperm count, acrosome integrity and membrane integrity of aging mice in the CD group were significantly decreased (*p* < 0.01), and the abnormal sperm rate was significantly increased (*p* < 0.01). Sperm motility, acrosome integrity and membrane integrity were significantly higher (*p* < 0.01) in the MR and HR groups than in the CD group, while the abnormal sperm rate was not significantly different between the CD, HR and MR groups. Unexpectedly, the sperm concentration and effective sperm count in the MR and HR groups were significantly lower than those in the CD group (*p* < 0.01, Figure 3). These results indicated that sperm concentration and sperm quality were reduced in aging mice, but methionine restriction could improve sperm quality to some extent but not sperm count, and methionine restriction from different sources had no significant difference in improving sperm quality.

### 3.4. Levels of Inflammation and Oxidative Stress in Testes of Aging Mice with Methionine Restriction

**Figure 4:**
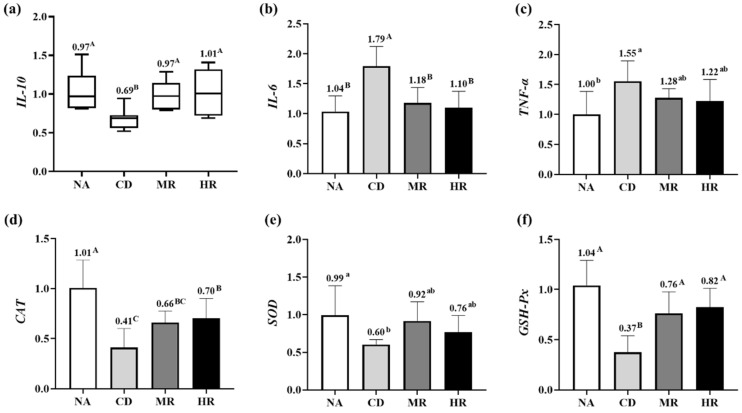
Relative mRNA expression of inflammation and oxidative stress-related factors in testes. Relative mRNA expression of (**a**) *Interleukin 10 (IL-10)*, (**b**) *Interleukin 6 (IL-6)*, (**c**) *Tumor necrosis factor α (TNF-α)*, (**d**) *Catalase (CAT)*, (**e**) *Superoxide dismutase (SOD)* and (**f**) *Glutathione peroxidase (GSH-Px)* in testes. Data for *IL-10* were non-normally distributed and expressed as median and 10–90% percentile, while data for the remaining indicators were normally distributed and expressed as mean ± SD (NA, CD, MR and HR groups, *n* = 8). In the same figure, values with different small letter superscripts indicate significant differences (*p* < 0.05), and values with different capital letter superscripts indicate extremely significant differences (*p* < 0.01).

As shown in Figure 4a–c, the detection results of testicular inflammatory factors showed that the relative mRNA expression of *IL-10* was significantly lower (*p* < 0.01) while the levels of *IL-6* (*p* < 0.01) and *TNF-α* (*p* < 0.05) were significantly higher in the CD group than in the NA group. Importantly, the relative mRNA expression of *IL-10* was significantly higher, whereas the level of *IL-6* in the MR and HR groups was significantly lower than that in the CD group (*p* < 0.01), and the levels of *IL-10* and *IL-6* in the MR and HR groups were not significantly different from those in the NA group (*p* > 0.05). These results suggest that the increased expression of pro-inflammatory factors (IL-6) and decreased expression of anti-inflammatory factors (IL-10) in aging mice can be reversed by methionine restriction.

The relative mRNA expression of oxidative stress-related factors showed that the levels of the antioxidant enzymes *CAT* (*p* < 0.01), *SOD* (*p* < 0.05) and *GSH-Px* (*p* < 0.01) were significantly decreased in the CD group compared with the NA group. The relative mRNA expressions of *CAT* and *GSH-Px* in the MR and HR groups were higher than those in the CD group, and there was no difference in the two indices between the MR and HR groups (Figure 4d–f). The results showed that the antioxidant capacity of the testes of aging mice was decreased and that methionine restriction from different sources could improve the antioxidant capacity of aging mice.

### 3.5. Methionine Metabolites in Testes of Aging Mice with Methionine Restriction

**Figure 5:**
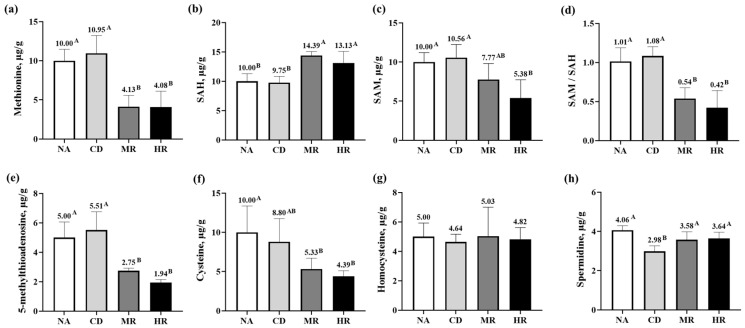
The contents of methionine metabolites in testes. The contents of (**a**) Methionine, (**b**) S-adenosylhomocysteine (SAH), (**c**) S-adenosylmethionine (SAM), (**d**) SAM/SAH, (**e**) 5-methylthioadenosine, (**f**) Cysteine, (**g**) Homocysteine, and (**h**) Spermidine in testes. Data are presented as the mean ± SD (NA, CD, MR and HR groups, *n* = 6). In the same figure, values with different capital letter superscripts indicate extremely significant differences (*p* < 0.01).

As shown in Figure 5, mice in the NA and CD groups were fed the same amount of methionine; only the spermidine content in the testes of mice in the CD group was significantly lower than that in the NA group (*p* < 0.01), and the contents of methionine, S-adenosylhomocysteine (SAH), S-adenosylmethionine (SAM), 5-methylthioadenosine, cysteine and homocysteine and the SAM/SAH ratio were not significantly different from those in the NA group (*p* > 0.05). The levels of methionine, 5-methylthioadenosine and cysteine and the SAM/SAH ratio in the testes of the MR and HR groups’ mice that were restricted to methionine were significantly lower than those of the CD group (*p* < 0.01), but the levels of SAH and spermidine were significantly higher than those of the CD group (*p* < 0.01). From these results, it can be concluded that methionine metabolites were mainly influenced by the dietary methionine level and were not related to the age of the mice. However, methionine-induced spermidine levels were affected by age and methionine restriction.

### 3.6. DNA and RNA Methylation Levels in Sperm of Aging Mice with Methionine Restriction

**Figure 6:**
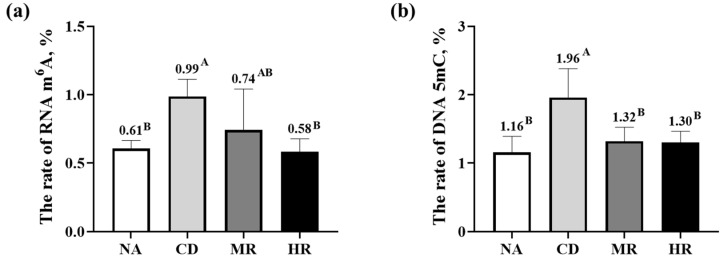
DNA and RNA methylation levels in sperm. (**a**) The rate of RNA N6-methyladenosine (m^6^A); (**b**) The rate of DNA 5-Methylcytosine (5mC). Data are presented as the mean ± SD (NA, CD, MR and HR groups, *n* = 6). In the same figure, values with different capital letter superscripts indicate extremely significant differences (*p* < 0.01).

The relative levels of RNA m^6^A and DNA 5mC in the CD group were significantly higher than those in the NA group (*p* < 0.01). The levels of RNA m^6^A and DNA 5mC in the HR group were significantly lower than those in the CD group (*p* < 0.01), but only the level of DNA 5mC in the MR group was lower than that in the CD group (*p* < 0.01, Figure 6). 

### 3.7. Correlation Analysis of Sperm Quality Parameters, Methionine Metabolite Level and Sperm RNA m^6^A and DNA 5mC Level in Aging Mice

**Figure 7:**
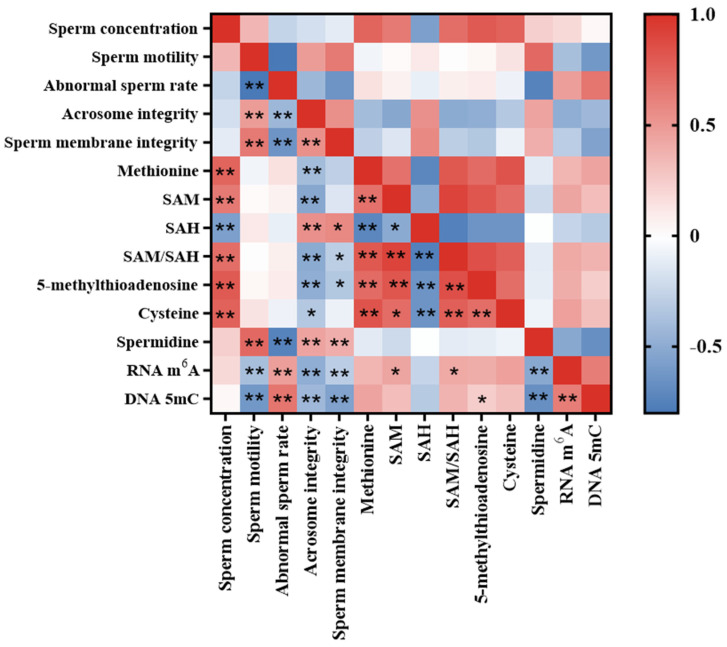
Correlation analysis of sperm quality parameters, methionine metabolite level and sperm RNA m^6^A and DNA 5mC levels in aging mice (*n* = 24). * Indicates a significant correlation between indicators, *p* < 0.05; ** indicates a very significant correlation between indicators, *p* < 0.01.

As shown in Figure 7, a correlation analysis of sperm quality parameters with methionine metabolite levels, sperm RNA m^6^A and DNA 5mC levels showed that sperm concentration was significantly positively correlated with methionine, SAM, SAM/SAH, 5-methylthioadenosine and cysteine (*p* < 0.01), while significantly negatively correlated with SAH (*p* < 0.01). Sperm motility, sperm acrosome integrity and membrane integrity were negatively correlated with sperm RNA m^6^A methylation and DNA 5mC methylation (*p* < 0.01) and positively correlated with spermidine content (*p* < 0.01), whereas abnormal sperm rate was related to methionine metabolites and sperm methylation in contrast to sperm motility. More importantly, sperm acrosome integrity and membrane integrity were also positively correlated with SAH content (*p* < 0.05) and significantly negatively correlated with SAM/SAH and 5-methylthioadenosine levels (*p* < 0.05). It was shown that low levels of methionine and sperm methylation were beneficial for improving sperm quality, especially sperm acrosome integrity and membrane integrity.

The relationship between methionine metabolites and sperm RNA m^6^A and DNA 5mC levels was analyzed. The results showed that testicular arginine levels were significantly negatively correlated with sperm RNA m^6^A and DNA 5mC levels (*p* < 0.01), whereas SAM and SAM/SAH ratio levels were significantly positively correlated with sperm RNA m^6^A levels (*p* < 0.05), and 5-methylthioadenosine levels were significantly positively correlated with sperm DNA 5mC methylation levels (*p* < 0.05). There was also a significant linear correlation between methionine metabolites.

## 4. Discussion

Senescence is an inevitable biological problem. The semen quality in male animals decreases with age in animals and damages its economic value. In order to explore nutritional strategies to delay the decline of semen quality caused by aging in male animals, this study took aging mice as model animals and studied the improvement effect of methionine restriction on the sperm quality of aging mice using L-methionine or HMTBA as the methionine source. The results of this study showed that methionine restriction from different sources alleviated the oxidative stress and inflammatory state of the testes of aging mice, which may reduce the level of sperm RNA m^6^A and DNA 5mC levels by changing the content of methionine metabolites, thus alleviating the decline of sperm quality in aging mice.

### 4.1. Effects of Methionine Restriction on Sperm Quality in Aging Mice

D-galactose is a reducing sugar that normally exists in the body. When the concentration of D-galactose exceeds normal levels, it is converted into aldehydes and hydrogen peroxide [28,29]. Animals injected with D-galactose exhibit aging-related phenotypes, such as neurological defects, decreased immune responses, reduced antioxidant enzyme activity and increased ROS production [28,30]. In our study, D-galactose-injected aging mouse models showed increased levels of testicular oxidative stress and inflammation, impaired testicular morphology and reduced sperm quality. This is similar to the performance of D-galactose-constructed aging mice reported by Liao et al. [31], suggesting that the aging mouse model was successfully constructed. Methionine restriction has demonstrated anti-aging effects in a variety of model animals; however, the effect of methionine restriction on the decline of sperm quality in animals is still unclear [32]. The current study found that methionine restriction from different methionine sources significantly increased sperm motility and morphological integrity but decreased sperm concentration in aging mice. This may be because methionine restriction may alleviate the impaired reproductive function caused by aging in male animals to a certain extent by reducing oxidative stress and inflammatory responses, changing methionine metabolism and sperm RNA m^6^A and DNA 5mC levels, but it cannot restore spermatogenesis in male animals. However, aging permanently affects spermatogenic function in male animals by damaging the testicles and epididymis [33,34]. This also explains why aging mice had lower sperm concentrations than normal mice at normal methionine levels. Overall, methionine restriction from different sources effectively improved effective sperm count and sperm quality in aging animals, although sperm concentration decreased. These results suggest that the application of methionine restriction may be an effective nutrition strategy to improve the sperm quality of aged male animals.

### 4.2. Effects of Methionine Restriction on Inflammation and Oxidative Stress in Testes of Aging Mice

Methionine restriction can alter the metabolism of methionine, and methionine metabolites, such as spermidine, GSH, sulfuretted hydrogen and taurine, all have antioxidant properties. Methionine restriction can reduce oxidative stress and inflammation by increasing the production of endogenous sulfuretted hydrogen through the transsulfuration pathway and reducing oxidative damage caused by mitochondrial reactive oxygen species [18]. Therefore, methionine restriction may alleviate organ damage caused by aging by increasing the content of antioxidant metabolites and reducing oxidative damage. In the current study, the exfoliated germ cells of aging mice fed with the methionine restriction diet were fewer and their structure was more complete. The relative mRNA expressions of inflammatory factors and oxidative stress-related factors in testicular tissues were significantly decreased, the expressions of male genes were up-regulated and the content of serum testosterone was up-regulated compared with those in the other groups. These results suggest that methionine restriction has a mitigating effect on testicular damage caused by aging, which is consistent with our observation regarding sperm quality in aging mice.

### 4.3. Effects of Methionine Restriction on Spermidine Content in Testes of Aging Mice

Polyamines are metabolites of methionine, which can participate in the regulation of aging by activating autophagy, and spermidine can also reduce the accumulation of free radicals in the body [35,36]. Aging reduces spermidine levels in mice due to the down-regulation of key genes related to the synthesis of spermidine [37]. However, methionine restriction could increase hepatic spermidine content by more than 25% in aging mice compared to a regular methionine diet [38]. Consistent with the conclusions of previous studies, our results also found that methionine restriction from different methionine sources significantly increased spermidine content in the testicular tissues of aging mice. The regulation of spermidine by methionine restriction may increase the transcription level of key genes in the spermidine synthesis pathway and thus promote the synthesis of spermidine [38]. The function of methionine restriction in regulating the synthesis of spermidine may be an important factor in alleviating the decline of sperm quality in aging animals.

### 4.4. Effects of Methionine Restriction on Sperm RNA m^6^A and DNA 5mC Levels in Aging Mice

Methionine metabolism in animals is a complex biochemical reaction system involving a variety of nutrients and enzymes, and its metabolites can regulate the body’s physiological functions. In the current study, methionine restriction with different sources decreased the sperm DNA 5mC level and RNA m^6^A level of aging mice by reducing the SAM, SAM/SAH ratio and 5-methylthioadenosine levels in testes. SAM is the primary product of the methionine transmethylation process, which is directly related to the concentration of methionine. Methionine restriction in animals means a lowered intake of methionine, and the contents of methionine and SAM also decrease [39]. Similar to the idea that methionine restriction affects the methylation state of the body by regulating the level of methionine metabolites, some researchers have found that calorie restriction affects aging by preventing changes in DNA methylation and inducing the expression of genes with anti-aging effects [40]. This suggests that methionine restriction may delay aging by affecting metabolite content and reducing RNA m^6^A and DNA 5mC levels, thus alleviating the decline in sperm quality in aging animals.

## 5. Conclusions

Methionine restriction from different methionine sources reduced the oxidative stress and inflammatory state of the testes and alleviated the decline of sperm quality in aging mice. The mechanism may be related to the change in methionine metabolite level and the inhibition of sperm DNA and RNA methylation.

## Figures and Tables

**Table 1:** Diet formulation for mice.

Ingredients	0.86% Methionine, %	0.17% Methionine, %	0.17% HMTBA, %
Amino acid mixture ^1^	7.80	7.80	7.80
L-methione	0.86	0.17	-
HMTBA ^2^	-	-	0.17
L-glutamic acid	-	0.69	0.69
Starch and sucrose	68.64	68.64	68.64
Soybean oil	8.00	8.00	8.00
Dextrin	5.00	5.00	5.00
Cellulose	5.00	5.00	5.00
Choline bitartrate	0.20	0.20	0.20
Mineral and vitamin mixture	4.50	4.50	4.50
Total	100.00	100.00	100.00

^1^ Amino acid mixture: L-arginine, L-lysine, L-histidine, L-leucine, L-isoleucine, L-valine, L-tryptophan, L-phenylalanine, L-threonine; ^2^ HMTBA: 2-hydroxy-4-(methylthio) butanoat.

**Table 2:** Primer sequences for RT-qPCR.

Gene Symbol	PCR Primer (5′-3′)
Steroidogenic acute regulatory protein (STAR)	AACGGGGACGAAGTGCTAAG CCGTGTCTTTTCCAATCCTCTG
Interleukin 10 (IL-10)	AGTCTTGGGCTACCTCC
	CTCGCTTCACTATCTGTCTG
Interleukin 6 (IL-6)	GGCGATGCGTCTAAGGAA
	GAAGGACTGGGCTGAAAATAAGGGGT
Tumor necrosis factor α (TNF-α)	TAGGCGAGGGAACAAGAG
	TGGGCAACCGAGTCATAC
Catalase (CAT)	GACAGATCAGATGAAGATGACTGGT
	GTGAGGAGGACAATTATTTCCAGTT
Superoxide dismutase (SOD)	TCAAGAATTCTGAAATGTGGAAGA
	GAGCAGAAGTTGCAGATCTTTTAGA
Glutathione peroxidase (GSH-Px)	AAGCTGTGCAAGGTGAGGAA
	CTTGATGGCATCGGTGATTT
GAPDH	CCTCCAAGGAGTAAGAGCC
	GTCTGGGATGGAAACTGG

## Data Availability

Data are contained within the article.
